# Reaching in reality and virtual reality: a comparison of movement kinematics in healthy subjects and in adults with hemiparesis

**DOI:** 10.1186/1743-0003-1-11

**Published:** 2004-12-14

**Authors:** Antonin Viau, Anatol G Feldman, Bradford J McFadyen, Mindy F Levin

**Affiliations:** 1 School of Rehabilitation, Faculty of Medicine, University of Montreal, Canada; 2 Center for Interdisciplinary Research in Rehabilitation (CRIR), 6300 Darlington, Montreal, Quebec, Canada; 3 Department of Physiology, University of Montreal, Canada; 4 Center for Interdisciplinary Research in Rehabilitation and Social Integration (CIRRIS), Department of Rehabilitation, Laval, Canada; 5 School of Physical and Occupational Therapy, McGill University, Canada

**Keywords:** arm reaching, prehension, rehabilitation, stroke, therapeutic approach, hemiplegia

## Abstract

**Background:**

Virtual reality (VR) is an innovative tool for sensorimotor rehabilitation increasingly being employed in clinical and community settings. Despite the growing interest in VR, few studies have determined the validity of movements made in VR environments with respect to real physical environments. The goal of this study was to compare movements done in physical and virtual environments in adults with motor deficits to those in healthy individuals.

**Methods:**

The participants were 8 healthy adults and 7 adults with mild left hemiparesis due to stroke. Kinematics of functional arm movements involving reaching, grasping and releasing made in physical and virtual environments were analyzed in two phases: 1) reaching and grasping the ball and 2) ball transport and release. The virtual environment included interaction with an object on a 2D computer screen and haptic force feedback from a virtual ball. Temporal and spatial parameters of reaching and grasping were determined for each phase.

**Results:**

Individuals in both groups were able to reach, grasp, transport, place and release the virtual and real ball using similar movement strategies. In healthy subjects, reaching and grasping movements in both environments were similar but these subjects used less wrist extension and more elbow extension to place the ball on the virtual vertical surface. Participants with hemiparesis made slower movements in both environments compared to healthy subjects and during transport and placing of the ball, trajectories were more curved and interjoint coordination was altered. Despite these differences, patients with hemiparesis also tended to use less wrist extension during the whole movement and more elbow extension at the end of the placing phase.

**Conclusion:**

Differences in movements made by healthy subjects in the two environments may be explained by the use of a 2D instead of a 3D virtual environment and the absence of haptic feedback from the VR target. Despite these differences, our findings suggest that both healthy subjects and individuals with motor deficits used similar movement strategies when grasping and placing a ball in the two reality conditions. This suggests that training of arm movements in VR environments may be a valid approach to the rehabilitation of patients with motor disorders.

## Introduction

Virtual reality (VR) is a computer-based, multisensory interactive simulation occurring at the same speed and time as events in the physical world. Different levels of immersion can be achieved ranging from complete 3D (cave, head-mounted display) to partial 2D (computer display, TV screen) with different hardware configurations. Interface devices (computer mouse, joystick, force sensor, cyberglove) allow the user to move in and interact with objects in the virtual environment. Of crucial relevance to rehabilitation is the potential for increasing the user's level of interaction with their real physical environment so as to maximize their return to community life [1]. The efficacy of using VR to retrain movement and the issue of whether training in a virtual environment will transfer to meaningful function in the real physical world has been explored in a number of studies with encouraging early results [2-4].

Neurophysiologists and rehabilitation specialists like physical and occupational therapists are beginning to be interested in VR as a tool to study motor control and to evaluate and treat motor deficits secondary to central nervous system lesions such as stroke [5]. The use of virtual computer-based interventions for telerehabilitation is also gaining in popularity because of the possibility of providing extended practice in the patient's own home or community environment [6,7]. The advantage of using VR in community, clinical and laboratory settings is that by virtue of its programmability, environments and the amount and type of feedback can be modified according to the user's motor capacities, motivation and therapeutic goals [5,8]. In addition, sensory parameters of the environment can be creatively adapted to evoke responses to a larger number of situations in a shorter amount of time than is available in physical set-ups. For example, in research studies, when determining the capacity to reach and grasp static targets, methodologies are often limited to one or two tasks because of the inability to easily adapt the experimental hardware. VR permits the use of more dynamic experimental set-ups in which object locations and orientations can be reliably and rapidly modified.

This study focused on the possibility of using virtual environments for the retraining of arm motor function in individuals with hemiparesis due to stroke. Major barriers to arm motor recovery after stroke are coordination deficits and the use of maladaptive movement strategies for reaching and grasping. Patient motivation and movement repetition are key factors in motor recovery [9-12]. Current practice of rehabilitation of reaching deficits after stroke is based on movement repetition of targeted tasks. However, improvements in tasks practised in clinical settings have not been shown to have adequate carry over into real world activities of daily living [10]. One of the factors that may decrease the real world relevancy of practice in the clinical setting is the lack of attention to the retraining of varied goal-directed, effector-relevant whole arm movements. VR is an ideal medium in which to create such practise environments that have the advantage of providing additional motivation to patients to perform repetitive movement and can be available in the home or community following formal rehabilitation [13]. Indeed, some studies have reported that motor gains achieved by patients with stroke in VR environments may transfer to physical tasks and be measurable using common clinical scales [2,5,14].

Despite the growing interest in the use of VR for motor retraining, it is not known if movements involving reaching and grasping objects in VR environments are performed in a manner similar to those done in the physical world. Thus, the goal of this study was to validate VR as a tool for studying reaching and grasping in healthy subjects and in individuals with hemiparesis by comparing movement kinematics of identical tasks made in a physical and a virtual environment. Since reaching and grasping deficits have been well characterized in individuals with hemiparesis [15-17], the purpose of the study was not to compare movements between groups but to establish the validity of using a VR environment for the study of movement in each group. Preliminary results have appeared in abstract form [18].

## Methods

Eight healthy subjects (4 males and 4 females; 56.8 ± 17.1 years) and 7 adults with hemiparesis (3 males and 4 females; 48.9 ± 18.6 years) with no prior experience with VR participated in the study. Potential participants were identified from discharge lists of Montreal area rehabilitation centres. Out of 17 medical charts screened, 12 patients met eligibility requirements according to study inclusion and exclusion criteria. Patients were included if they were under 60 years old, had sustained a single, non-traumatic unilateral stroke in the territory of the left middle cerebral artery and had arm paresis (3/7 for the hand and 6/7 for the arm on the Chedoke-McMaster Stroke Assessment Scale [19] (Table 1). Patients were excluded if they had cerebellar or brain stem lesions, shoulder pain or other neurological/orthopaedic conditions affecting reaching ability, visual field deficits, uncorrected problems of visual acuity or severe perceptuo-cognitive deficits (heminegligence, ataxia, receptive aphasia) determined by standard clinical tests. Of these 12 individuals, 10 expressed willingness to participate. After obtaining informed consent approved of by the institutional Ethics Committee, they were assessed by a physical therapist and 3 individuals were excluded because of inability to perform the task. Healthy subjects were recruited from the community. They had no orthopaedic or neurological disease. All patients had been discharged from all in- or out-patient clinical services.

**Table 1:** Demographic characteristics and clinical scores of participants with hemiparesis

Subject	Age (yrs)/sex	Time since injury (months)	Type of lesion	CM: arm	CM: hand
1	63/M	28	Temporo-parietal	6	6
2	42/F	34	Parietal	7	6
3	27/F	63	Parietal	7	3
4	51/F	51	Frontal	7	6
5	31/F	33	Temporo-parietal	6	6
6	47/M	64	Fronto-temporo-parietal	7	6
7	81/M	33	Temporo-parietal	7	6
Mean ± SD	48.9 ± 18.6	43.7 ± 15.3		6.7 ± 0.5	5.6 ± 1.1

M = male, F = female, CM = Chedoke-McMaster stroke assessment

**Figure 1:**
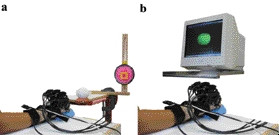
Experimental set-up. Physical (**a**) and virtual reality condition **(b)**.

Subjects performed 6 trials each of two near identical tasks set in the physical world or in a virtual environment. In both tasks, seated subjects grasped a real or virtual ball of 7 cm diameter with their right hand, beginning from the edge of a real or virtual table, reached forward by leaning the trunk and then placed the ball within a 2 cm × 2 cm yellow square on a real or virtual target (Figure 1). Care was taken to set-up the physical task so that the initial position of the arm, ball, table and wall were identical to that of the virtual task. Thus, in both environments, the initial position of the arm was about 0° flexion, 30° abduction and 0° external rotation (shoulder), 80° flexion and 0° supination (elbow) with the wrist and hand in the neutral position. The fingers were slightly flexed. The initial position of the ball was 13 cm in front of the right shoulder, 7 cm above and 3 cm to the left of the subject's hand. The target was placed 31 cm in front of the shoulder, 12.5 cm above and 14 cm to the right of the initial position of the ball (Figure 1).

For the VR task, the ball appeared on a computer screen inside a cube that also displayed the position of the subject's hand. The VR target was the upper right back corner of the cube. Subjects had to grasp the virtual ball, transport it to the VR target and release it. The VR environment was displayed in 2 dimensions (2D) on a computer screen placed 75 cm in front of subject's manubrium (Figure 1). The virtual representation of the subject's hand was obtained using a 22 sensor fibre optic glove (Cyberglove, Immersion Corp.) and an electromagnetic sensor (Fastrak, Polhemus Corp.) that was used to orient the glove in the 2D environment. Data from these devices were synchronized in real time. To enable the subject to "feel" the virtual ball, a prehension force feedback device (Cybergrasp, Immersion Corp.) was fitted to the dorsal surface of the hand. The Cybergrasp delivered prehension force feedback in the form of extension forces to the distal phalanxes of the thumb and each finger. Forces applied to the fingers were calibrated for each subject while he/she was wearing the Cyberglove. These ranged from 6 to 8 N per finger and all subjects perceived that they were holding a spherical object in their hand.

Prior to data collection, all participants practised the tasks in physical and virtual conditions (20 – 40 min). To better compare the participants' performance in the two environments, the glove and grasp devices were worn on the hand in both conditions (Figure 1).

Kinematic data from the right arm were recorded with 6 infrared-emitting diodes (IREDs) placed on the distal phalanx of the index and thumb, the distal head of the first metacarpal, the radial styloid process, the lateral epicondyle of the humerus and the acromion (120 Hz, Optotrak Motion Analysis System, Northern Digital Corp.).

Each trial was divided in two phases: 1) reaching and grasping the ball and 2) ball transport and release. For the first movement phase, 4 temporal and 4 spatial parameters of reaching and grasping were determined. Temporal parameters were movement time, time to peak wrist velocity (RPV), time to maximal hand aperture (RMGA), and the delay between them (RPV-RMGA). Spatial parameters were endpoint path curvature, maximal grip aperture, angular ranges of joint motion and elbow-shoulder interjoint coordination [16]. For the second movement phase, we determined one temporal (movement time) and 4 spatial (endpoint path curvature, trajectory length, angular ranges of joint motion and interjoint coordination) parameters.

Movement onsets and offsets of each phase were defined as the times at which the tangential velocity of the IRED on the index finger surpassed and remained above or fell and remained below 10% of the maximal peak velocity respectively. The temporal parameters (time to peak wrist velocity, time to maximal grip aperture) were normalized to movement time and the delay between them was calculated. For the spatial parameters, the curvature of the trajectory of the IRED on the index finger was estimated as the ratio between the actual trajectory length and a straight line segment between the initial and final positions [20]. Joint angular excursions were expressed as the difference in degrees between the angle at the beginning and the end of movement, according to movement times defined above. For interjoint coordination, we determined the slopes between elbow extension and 1) shoulder flexion and 2) shoulder abduction. The slope of the angle-angle relationship describes the relative contribution of each joint throughout the movement where a slope of 1 indicates an equal contribution of each joint. Slopes greater than 1 indicated a larger contribution of elbow extension than shoulder movement and vice versa. The relationship between both angles was considered linear since all regression correlation coefficients were = 0.8. However, since a linear approximation was used, the slope provides only a general estimate of the contribution of each angle.

### Statistical analysis

Both parametric and non-parametric statistics were used. For within-group comparisons between the two conditions of reality, Student t-tests were used. However, since variances were not homogeneous (Levene's test) for healthy subjects and participants with hemiparesis, non-parametric tests were used for between-group comparisons (Kruskal-Wallis ANOVA). A significance level of *p *< 0.05 was used, adjusted for multiple comparisons by type using the Bonferroni correction.

## Results

**Figure 2:**
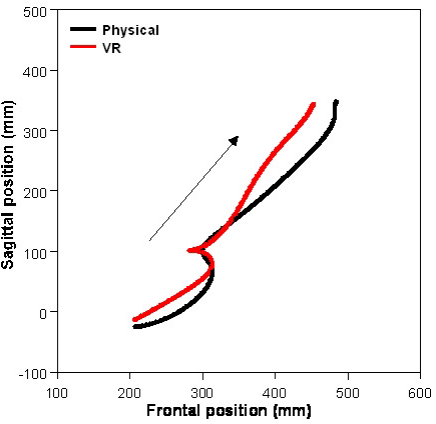
Mean endpoint (marker on the index finger) trajectories for the two phases of the movement task for one healthy subject in the two reality conditions.

All healthy subjects and participants with mild upper limb motor deficits were able to reach, grasp, transport, place and release the virtual ball using movement strategies that were similar to those used for the physical ball (Tables 2 and 3). Arm movement trajectories (Figure 2) were smooth and followed similar paths for movements made in both environments for both subject groups. Trajectory lengths were similar in both conditions for healthy subjects (289 ± 28 mm in real compared to 302 ± 55 mm in VR) and for participants with hemiparesis (251 ± 25 mm in real compared to 260 ± 30 mm in VR).

**Table 2:** Comparisons between reality conditions for the first phase of movement: reaching and grasping the ball.

	Healthy				Stroke			
	Physical condition		VR condition		Physical condition		VR condition	
	Mean	SD	Mean	SD	Mean	SD	Mean	SD
**Temporal parameters**								
Movement time – onset to grasping (s)	0.68	0.17	0.95	0.35	1.23†	0.27	1.43†	0.41
RPV (%)	44.9	10.4	41.8	6.1	34.3	12.6	40.8	2.9
RMGA (%)	72.5	12.5	60.9	11.8	73.5	16.1	65.3	9.1
Delay between RPV and RMGA (%)	31.6	16.6	19.2	10.7	34.5	15.0	24.3	8.1

**Spatial parameters**								
Curvature index	1.39	0.16	1.62	0.44	1.76	0.62	1.97	0.86
Wrist extension at grasping (°)	1.4	9.1	-3.9	8.2	12.1	2.2	4.5	14.1
Slope elbow extension/shoulder flexion	0.70	0.34	0.60	0.26	0.53	0.33	0.47	0.27
Slope elbow extension/shoulder abduction	2.30	2.02	2.31	2.54	2.65	1.66	2.30	1.26
Maximal grip aperture (mm)	95.7	16.4	90.2	20.5	89.8	20.2	84.9	19.3

RPV = relative time to peak velocity of the wrist; RMGA = relative time to maximal grip aperture; VR = virtual reality

† Significant difference between groups, Kruskal-Wallis, p < 0.05

**Table 3:** Comparisons between reality conditions for the second phase of movement: ball transport and release

	Healthy				Stroke			
	Physical condition		VR condition		Physical condition		VR condition	
	Mean	SD	Mean	SD	Mean	SD	Mean	SD
**Temporal parameters**								
Movement time – onset to placing (s)	0.84	0.29	1.18	0.31	1.49†	0.45	2.28†	0.82

**Spatial parameters**								
Curvature index	1.14	0.03	1.23	0.29	1.37†	0.27	1.42	0.57
Wrist extension at placing (°)	18.2	12.1	4.0*	8.1	20.3	8.2	6.4	16.0
Elbow extension (°)	25.6	6.9	38.4*	10.9	26.9	11.0	37.3	19.6
Shoulder flexion (°)	24.8	5.2	33.0	7.1	28.4	4.9	35.5	18.2
Shoulder abduction (°)	13.6	5.2	16.4	4.6	18.5	6.9	20.7	8.9
Slope elbow extension/shoulder flexion	1.08	0.08	1.21	0.16	1.01	0.26	1.18	0.15
Slope elbow extension/shoulder abduction	2.36	0.76	2.88	1.19	1.51†	0.69	1.48†	0.51

* Significant difference between reality conditions, Student t-tests with Bonferroni correction (p < 0.05/4 angles = 0.013); VR = virtual reality

† Significant difference between groups, Kruskal-Wallis, p < 0.05

**Figure 3:**
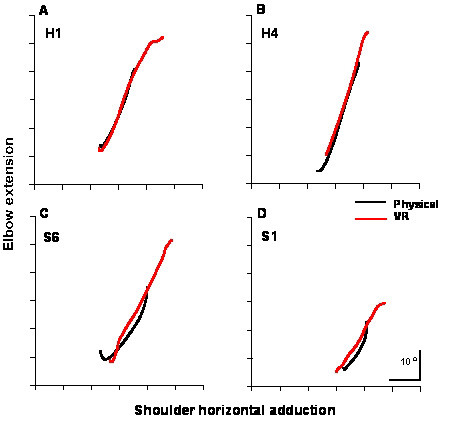
Interjoint coordination. Relationship between elbow extension and shoulder horizontal adduction (mean traces per condition) during the second phase of the movement (placing) for both conditions in two healthy subjects (**A,B**) and in two individuals with hemiparesis (**C,D**). In all examples, subjects used more elbow extension in the virtual reality condition.

In healthy subjects, the temporal and spatial aspects of the two phases of the task were almost identical between the physical and virtual conditions (Tables 2, 3). However, there was a non-significant tendency to make movements more slowly and to use less wrist extension for grasping during the first phase of the movement (reaching and grasping the ball) in the virtual condition (Table 2). During the second phase, healthy subjects used significantly less wrist extension (paired t-test, p < 0.05) and more elbow extension (paired t-test, p < 0.05) to place the ball on the virtual vertical surface (Table 3, Figure 3). In these subjects, there were no other differences between any other temporal or spatial parameter for both movement phases (peak wrist velocity, relative time to peak wrist velocity, timing of maximal grip aperture, trajectory curvature or interjoint coordination).

Movements made by individuals with hemiparesis in the physical environment differed from those made by healthy subjects in three ways. In both phases, movements were significantly slower and in the second phase, trajectories were more curved and interjoint coordination was altered (Tables 2 and 3). In particular, the slope of the relationship between elbow extension/shoulder abduction was lower than in healthy subjects during the second phase of the movement (p < 0.02, Figure 3). This decrease in slope was due to a more abducted position of the shoulder in the patient group. Despite these differences, patients showed tendencies similar to healthy subjects when reaching and grasping in the VR environment compared to the real environment (Tables 2, 3). They tended to decrease the speed of movements made in VR compared to the physical environment, to use less wrist extension in both movement phases and to use more elbow extension in the second phase of the movement. In addition, 5 out of 7 participants with hemiparesis significantly decreased the wrist extension while 4 increased elbow extension at the end of the second phase of the movement (at the time of placing the ball) in the VR condition.

## Discussion

The similarity in movement kinematics between physical and virtual reaching and grasping suggests that virtual reality may be an effective environment for rehabilitation. Interest in training in virtual environments is increasing amongst rehabilitation professionals in light of recent evidence suggesting that neuronal recovery after stroke-related brain damage critically depends on the motivation of the individual and the intensity of training [10,11]. Virtual reality represents a novel training environment in which a wide variety of tasks can be easily practiced. It is also becoming increasingly accessible with the advent of home-based computers and telerehabilitation technology [7]. Thus, the demonstration that movements practiced in a virtual environment are kinematically similar to movements with physical objects is essential to ensure the transfer of training benefits to the real-life situation.

Our results show that subjects tended to decrease wrist extension and increase elbow extension in the virtual compared to the physical condition. Two principal factors may explain those differences: the absence of depth perception in the VR condition and the absence of tactile feedback at the end of the reach.

Binocular vision enables humans to perceive depth in a 3 dimensional (3D) environment. This faculty is called stereopsis (for a review see [21]). Since the VR condition in our experimental set-up was a 3D task presented on a 2D display, the results can be compared with those investigating reaching and grasping movements made under conditions of monocular vision in which depth perception is reduced. Indeed, such studies have shown that reaching and grasping movements are characterized by shorter movement time and shorter relative time to maximal grip aperture [22].

The difference in depth perception in the 2D virtual environment may also be responsible for the tendency to increase elbow extension in both groups. Previous studies have shown that fine motor corrections are produced by distal joints [23]. The 2D display resulted in the subject underestimating the real distance to the wall so that during the course of the second phase of the movement, the subject had to compensate by increasing the extension of the limb until the screen display indicated that the ball had reached the target distance. This caused a slight change in strategy for the second phase of the movement requiring an increase in the amount of elbow extension. Participants with hemiparesis showed the same tendencies as the healthy group but differences were not significant due to subject variability. Changes in motor patterns may be avoided by using 3D immersive environments, such as those visualized through a head-mounted display.

The absence of depth perception cannot explain the decrease of wrist extension at the end of the second phase of the movement in VR compared to the physical condition. A more likely explanation involves the type of haptic feedback provided to the subject. In the physical condition, subjects had to extend the wrist so that the ball and not their fingers would make contact with the target. In the VR condition, wrist extension was not necessary because subjects only had to place the ball at the coordinates of the virtual wall without encountering a physical barrier. To avoid such differences between physical and VR environments, relevant haptic feedback is necessary to indicate contact of the hand with the object or target. Another way is to integrate physical objects into the VR environment such as the manipulation of a real paper envelope in real-time in a VR environment [6], or the mimicking of irregularities in a walkway with a multi-dimensional movement platform [24,25].

Overall, the finding that both healthy subjects and individuals with motor deficits used similar movement strategies in a physical and a limited virtual environment suggests that VR technology is a valuable tool for studying and retraining reaching, grasping and placing movements. Whether movement kinematics may be improved with the use of interfaces providing stereopsis and more relevant haptic feedback should be investigated by comparing movements made in immersive to those made in non-immersive virtual environments.
